# An Algorithm for Automatic Generation and Evaluation of Leaflet Apparatus Models for Heart Valve Prostheses

**DOI:** 10.17691/stm2022.14.4.01

**Published:** 2022-07-29

**Authors:** P.S. Onischenko, K.Yu. Klyshnikov, Е.А. Ovcharenko, L.S. Barbarash

**Affiliations:** Junior Researcher, Laboratory of New Biomaterials, Department of Experimental Medicine; Research Institute for Complex Issues of Cardiovascular Diseases, 6 Sosnovy Blvd, Kemerovo, 650002, Russia; Researcher, Laboratory of New Biomaterials, Department of Experimental Medicine; Research Institute for Complex Issues of Cardiovascular Diseases, 6 Sosnovy Blvd, Kemerovo, 650002, Russia; Head of the Laboratory of New Biomaterials, Department of Experimental Medicine; Research Institute for Complex Issues of Cardiovascular Diseases, 6 Sosnovy Blvd, Kemerovo, 650002, Russia; Professor, Academician of the Russian Academy of Sciences, Chief Researcher; Research Institute for Complex Issues of Cardiovascular Diseases, 6 Sosnovy Blvd, Kemerovo, 650002, Russia

**Keywords:** heart valve prosthesis, simulation, algorithm for generation and evaluation of leaflet apparatuses

## Abstract

**The aim of the study** is to develop and verify an algorithm for automatic generation of leaflet apparatus models for prosthetic heart valves, to optimize the basic parameters of the models in order to minimize the stress-strain state and maximize the geometric area of the orifice.

**Materials and Methods:**

The suggested algorithm consists of three blocks: “Generator”, “Modeling”, “Analysis”. The first block creates a three-dimensional model of the leaflet apparatus using the specified parameters (height, radius, thickness, degree of “sagging”, angle of the free edge deviation). Numerical simulation of the apparatus functioning is further performed using the finite element method. Then, the statistical analysis of the von Mises stresses is done and the opening area of the design in question is calculated.

Verification was performed by comparing quantitatively the lumen areas of the leaflet apparatus in the open state, obtained from the literature data for the Trifecta bioprosthesis (19, 21, and 23 mm in diameter), with the results of the described algorithm operation.

**Results:**

The verification of the algorithm has demonstrated the following deviations in the lumen area in the open state: 2.85% for 19 mm, 14.81% for 21 mm, and 23.17% for 23 mm models. This difference is due to the choice of the model material (no data could be found on the physical and mechanical properties of the pericardium used for the fabrication of the Trifecta bioprostheses).

The generation of a large number of designs (n=1517) without fixation of certain geometry parameters has shown that thickness of the leaflet apparatus makes the greatest contribution to the degree of opening; its dependence on the thickness and arising peak von Mises stresses has been demonstrated. Of the valvular models obtained, 278 showed the opening degree greater than 80% and maximum peak von Mises stresses below 4 MPa for the proposed model of the pericardium, which is 65% below the ultimate strength of the material.

Out of 278 leaflet models, 3 “optimal” designs were selected meeting the diameter criteria of 19, 21, and 23 mm. The loss index for them was 0.24, 0.19, 0.20 with the opening degrees of 88.28, 84.48, 88.12%, and maximum peak von Mises stresses of 3.62, 1.21, 1.87 MPa, respectively.

**Conclusion:**

The developed algorithm makes it possible to automatically generate three-dimensional models of the leaflet apparatus, numerically simulate the opening process using the finite element method, statistically analyze the results obtained, and calculate the lumen area. The algorithm was verified based on the data for the Trifecta bioprosthesis of three standard sizes. The presented algorithm can be used both for the research and development of various designs and for obtaining “optimal” models of sash devices.

## Introduction

High prevalence of cardiovascular diseases, acquired valvular defects in particular, results in the increase of operations for their replacement both in Russia (about 10 thousand operations annually [1]) and in the world (over 250 thousand [1, 2]). In contrast to mechanical prostheses, the biological ones do not require anticoagulant therapy, provide hemodynamics similar in character to the native, and can be implanted by a transcatheter technique. However, despite these benefits [3, 4], 50% of the implanted prosthetic heart valves develop structural changes in 10–15 years (leaflet rupture, calcinosis, pannus formation, leaflet avulsion in the commissural zone), which impair their functions [5, 6]. These alterations are caused by a prolonged effect of the immune system (immune rejection) on the implant [7] and accumulated mechanical fatigue damage in the leaflet apparatus [6, 8].

To solve the problem of the immune response, new materials are actively being developed [9, 10], methods of biomaterial conservation and treatment are being studied [11-13]. Engineers and researchers also pay attention to the second aspect of prosthesis dysfunction, fatigue damage, in order to create new types of heart valve prostheses without these drawbacks. However, their development is a great and time-consuming task at every step beginning from the design and material choice up to the grounding the safety and durability of the finished product [14]. It is unknown which of the characteristics of the implant makes the greatest contribution to the total strength of the medical device. The authors of some works demonstrate a significant effect of stress field distribution on the risks of damage to the prosthetic biomaterial, exposed to a long-term functioning, both in numerical [15, 16] and natural investigations [17] recommending optimization of a leaflet design in order to reduce the amplitude of stresses. Frequently, the current approaches to such optimization of the leaflet apparatus are of a subjective or exploratory character. Thus, a “classic” method of designing the leaflet apparatus for heart valve prostheses consists in the iterative selection of the optimal geometric parameters in the cycle: designing the “initial” geometry — hydrodynamic testing *in vitro*; correction of the leaflet geometry — retesting, and so on. The current tools of design and engineering analysis make it possible to simplify and reduce this succession by computer simulation [15, 18, 19] and exclusion of ineffective leaflet models. However, the first stage of forming the “initial” geometry remains unchanged and bears the burden of subjective conception of designers/ technologists, which may be originally incorrect.

A proposed option to reduce the probability of dysfunctions consists in optimization of the geometry of the most mobile and important element in the valve, its leaflet apparatus, which experiences the greatest loads and deformations during the cardiac cycle. In recent time, the researchers in different countries have attempted to develop the leaflet apparatus [20-23] using a more systemic approach to design based on the mathematical methods and algorithms of geometry optimization excluding the above-mentioned subjectivity. This approach was motivated by a wider choice of the “initial” forms of the leaflet apparatus, introduction of the qualitative characteristics of their selection, search for design–effectiveness interconnections for a given component in the perspective of developing more perfect models of prosthetic heart valves. However, such works have some disadvantages: parametrized lists of linear dimensions create difficulties in some cases for understanding differences between the models when only one parameter is changed; researchers use maximum stress as the only parameter characterizing the functionality of a specific prosthesis design; the technologies of simulating the liquid–solid interaction are rather time-consuming (as they need computation of liquid movement apart from the deformation of a solid body) and cannot be operatively used in clinical practice.

The proposed algorithm for designing and evaluating the models of the leaflet apparatus for heart valve prostheses allows one to create final geometries with clear and comprehensible characteristics, automatically perform numerical simulation of the functioning process with the possibility of using certain material properties and setting pressures acting on the leaflet from the aorta and left ventricle. Linear dimensions may be obtained by processing patient’s CT images and a pressure gradient from the results of Doppler echocardiography [24].

**The aim of the study** is to describe the developed algorithm for automatic generation of the leaflet apparatus models for heart valves, their numerical analysis and processing of the results obtained; to generate a large number of designs and choose the optimal ones for the prostheses with diameters of 19, 21, and 23 mm.

## Materials and Methods

### Algorithm for automatic generation of the leaflet apparatus

The algorithm for generation and investigation of the leaflet apparatus models proposed in the present work represents a succession of numerical methods including three blocks:

1. Generator. The task of this block is to create a three-dimensional facet leaflet model in STL format on the basis of the geometric input data. Owing to the automatic realization, it is possible to generate a large file of 3D models varying input data within the specified ranges.
2. Simulation. The work of every 3D leaflet model created by the generator is numerically simulated. This stage uses the final element method to analyze the stress-strain state in the engineering analysis environment.
3. Analysis. The quantitative characteristics of the numerical simulation are automatically analyzed: the stress-strain state and effectiveness of the work of every simulated leaflet.

A detailed description of each stage is presented below.

#### “Generator” block

The developed algorithm receives geometric parameters of the designed leaflet apparatus from the user in a simple and understandable form: height, size, degree of leaflet dome sagging, its thickness, and angle of the free edge deviation. On the basis of the point cloud generated by these parameters, building of the leaflet node links in a single closed surface is performed. Then, a 3D mesh is built and files are created, which will be used for initiating a numerical experiment (see “Simulation” block). The “Generator” is realized in the form of its own algorithm using the package of applied programs Matlab R2021a (The MathWorks, USA) for solving the tasks of technical computations.

**Figure 1.**
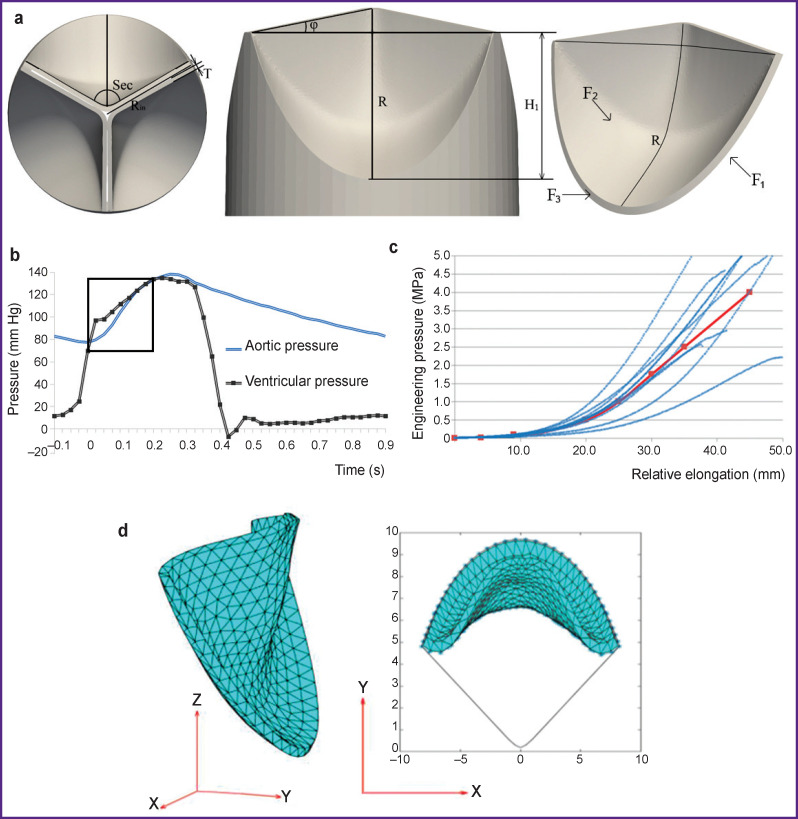
Materials and methods of investigation: (а) designations of parameters for building the geometry and surface to which boundary conditions were applied; (b) pressure profiles where aortic pressure is applied to the F2 surface and ventricular to F1; the phase of the leaflet apparatus opening is marked by a rectangle; (c) results of testing the pericardium samples on the universal testing machine; the red line shows material properties selected for numerical simulation; (d) a graphic presentation of the deformed leaflet apparatus (left) and the top view (right); grey line shows the leaflet contour in the initial position

The following set of geometric parameters is used as input data (Figure 1 (a)):

H_1_ (mm) — height of the proposed leaflet from its lower part up to the top point of the commissural strut;

φ (°) — angle of the free leaflet edge deviation;

R (%) — degree of leaflet dome “sagging”;

R_in_ (mm) — radius of the proposed prosthesis;

T (mm) — leaflet thickness;

Sec (°) — the number of degrees occupied by one leaflet.

#### “Simulation” block

The opening phase of the proposed prosthesis has been considered within the frames of this work, which allowed us to neglect the possible contacts and to simplify the model up to the investigation of one leaflet. The files prepared on the generator were transmitted to the engineering analysis environment Abaqus/CAE (Dassault Systemes, France) in which the model leaflet opening process was automatically numerically analyzed.

Boundary conditions for numerical simulation were set for the surfaces (see Figure 1 (a)) within the time segment of 0–0.2 s (Figure 1 (b)), which corresponds to the phase of leaflet apparatus opening:

F_1_ — pressure from the left ventricle (Figure 1 (a), ventricular pressure);

F_2_ — pressure from the aorta (Figure 1 (a), aortic pressure);

F_3_ — part of the leaflet apparatus fixed from the motion in 3 degrees of freedom in cylindrical coordinates.

The model of the material for numerical simulation was obtained by testing the samples (n=10) of xenopericardium, from which a leaflet apparatus of clinical biological prostheses was fabricated, on the universal Z-series testing machine (Zwick/Roel, Germany) using the sensor with a nominal load of 50 N at the ambient temperature of 37°С maintained by a thermal chamber. Samples were cut on the cutting press using a knife of a specific shape (ISO 37, type 4). Sample thickness was measured with a TRP thickness gauge with ±0.01-mm margin of permissible error (pressing force below 1.5 N). The speed of the cross-arm movement during testing was 20 mm/min. The obtained data were exported as the ratio of the relative elongation (mm) to stress (MPa) endured by the sample.

Taking into consideration the variability of the biomaterial mechanical properties determined by its natural non-uniformity, the whole set of separate curves was averaged using a third-order polynomial function built by the method of normal equations. In this way, a stress-strain conditional average curve was obtained for this material, which was employed at the stage of numerical simulation. Goodness of fit degree between the approximating curve and initial data of the uniaxial tensile was estimated by the coefficient of determination R^2^, which for the third-degree polynomial was equal to 0.86, which may be considered satisfactory for the tasks of the present study. The resulted properties of the material being used further for numerical simulation are shown with a red line in Figure 1 (c).

#### “Analysis” block

Using the results of numerical simulation, the mean value, maximum von Mises stress, and standard deviation were calculated. The degree of opening (DO) of the leaflet apparatus was computed as a ratio of a leaflet area projection on the XY surface to the initial non-deformed state (Figure 1 (d)). Since the considered model contains one leaflet, the area of its projection in the non-deformed state will occupy 1/3 of the circumference area with the R_in_ radius. Consequently, the desired DO of the leaflet apparatus may be determined by the formula:

DO=SScirc/3⋅100%,

where *S*_circ_ is the area of the circumference with the R_in_ radius; *S* is the area of opening during simulation.

To evaluate integrally the designs under consideration, a loss index (LI) was introduced, the formalized form of which may be written down in the following way:

LI=(100%−DO)2+(δlim−δmax)2,

where d_max_ — maximum stress arising in the process of simulation; d_lim_ — ultimate strength of the pericardium [25]. A simple interpretation may be presented by “the less, the better” expression. At DO=100% and zero maximum stresses, the LI will be equal to 0, in other cases it will tend to 1. At the increased values of d_lim_, the proposed method of assessment will show the value exceeding 1, which also demonstrates a failure of the considered leaflet apparatus design.

### Algorithm verification

**Figure 2.**
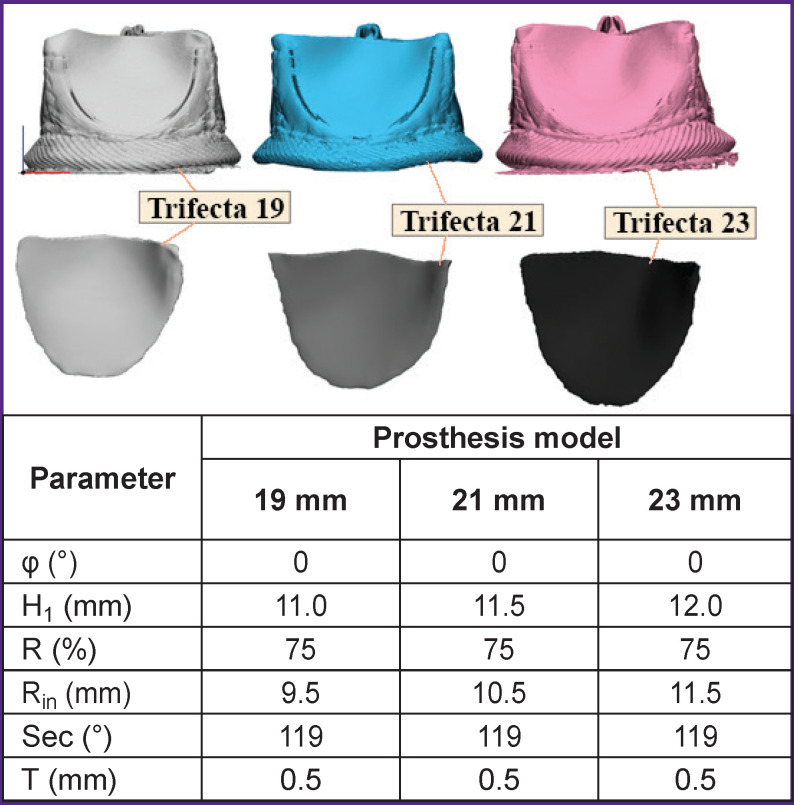
Three-dimensional reconstructions of clinical heart valve prostheses (three standard sizes of Trifecta prosthesis) obtained on the basis of microtomographic analysis, and quantitative measurements of their geometric parameters

Despite the application of the validated engineering tools, i.e. building faceted bodies and numerical simulation on the Abaqus/CAE platform, the generation− simulation−analysis integral link requires quality and consistency testing. In this connection, generation of three leaflet geometries of the Trifecta commercial clinical prosthesis, 19, 21, and 23 mm in size (St. Jude Medical, Inc., USA) on the basis of changing the geometric characteristics of highly precise 3D models using the proposed algorithm became a separate block for investigation (Figure 2). These models were obtained by microtomographic scanning using the OREL-MT experimental system (Tomsk, Russia). Using the obtained two-dimensional DICOM slices, facet bodies were reconstructed and their parameters measured (see the table in Figure 2).

These measurements of the 3D models were used as input data for the generation of three leaflets by means of the developed algorithm for each of the presented Trifecta bioprostheses at the stage of validation. The literature data were used as a material model for this case [26], since Trifecta bioprostheses are fabricated from the bovine xenopericardium stabilized with glutaraldehyde. The area of leaflet opening obtained during simulation by the proprietary algorithm was compared with the data from the experimental studies reported in the literature [27-29] and manufacturer’s documentation for this bioprosthesis [30].

### Investigation of a large set of leaflet geometries

The second step of our work was an attempt to assess the leaflet apparatus varying the parameters, the range of which was chosen to cover the majority of the existing tricuspid valve prostheses in aortic and tricuspid position. 1517 unique geometries were generated by our algorithm. They became the basis of numerical simulation. The following boundaries for changing the leaflet apparatus parameters were set:

H_1_=[10; 25] mm;

φ=[–30; 30]^°^;

R=[0; 100]%;

R_in_=[7.5; 20.0] mm;

T=[0.1; 1.0] mm.

## Results

### Verification

**Figure 3.**
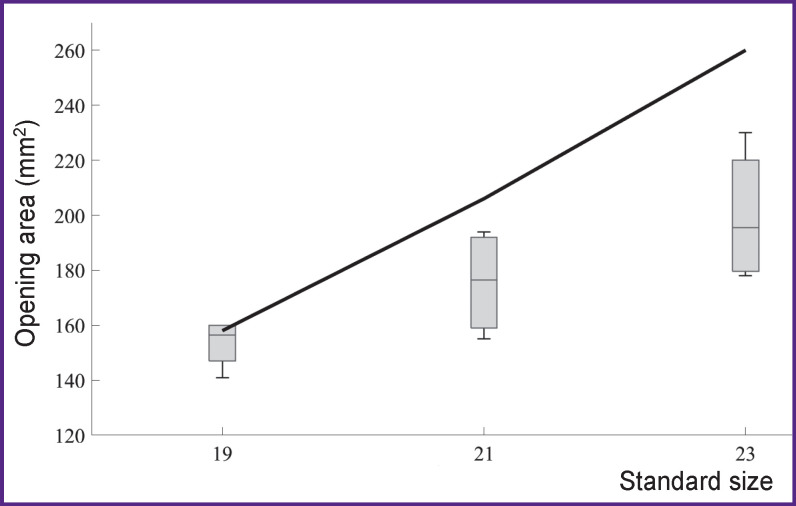
Comparison of the values of opening areas for the leaflet apparatus models obtained in the process of numerical simulation using the proposed algorithm (*black line*) with the data derived from the literature sources [27-30] The results are presented as median, 25 and 75 percentiles, minimum and maximum for each standard size

In the process of verification, DO of the built algorithms for leaflet apparatus models were evaluated and the results obtained were compared with those of the clinical studies [27-29] and with the manufacturer’s documentation [30] (Figure 3).

It has been established that the algorithm is capable of reproducing the process of opening the 19-mm Trifecta prosthesis with the error of 2.85%, 21-mm prosthesis — 14.81%, and 23-mm — 23.17% according to the “area of leaflet apparatus opening” parameter. These differences are caused by the model of the material, which is “softer” than the one used in the fabrication of the reference prosthesis [26].

### Investigation of a large set of leaflet geometries

**Figure 4.**
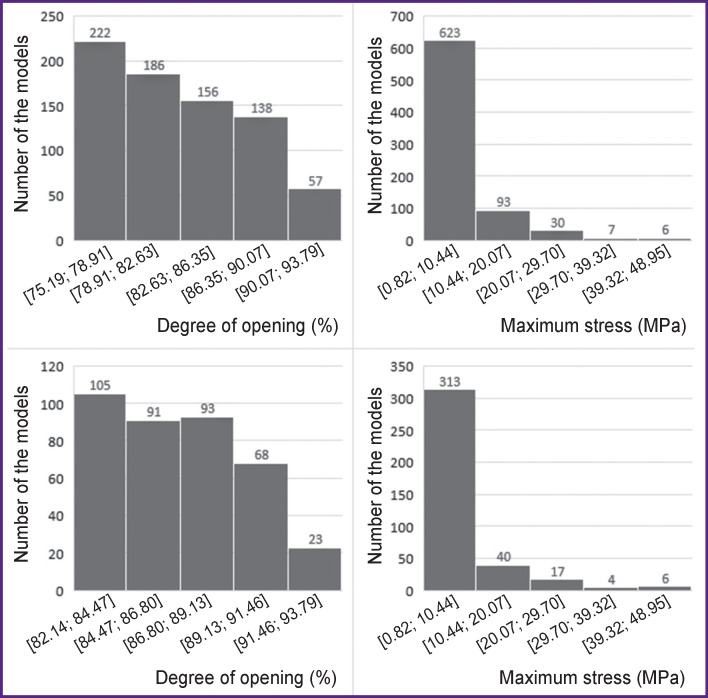
Quantitative distribution of models by the “degree of opening” and “maximum stress” parameters: Q3 and Q4 — from above, Q4 — from below

Analyzing the results of 1517 models built, a significant scattering of the DO parameter (71.97±15.91%) has been noted. So, for 759 models (Q3 and Q4, DО>75.19%) the mean DO value was 82.68±4.8% and maximum arising stress — 6.25±7.28 MPa. In the “best” 380 leaflet apparatuses (Q4, DО>82.14%), this value was 86.76±2.96% with maximum stress of 6.71±8.08 MPa. The histograms of distribution by the degree of opening and maximum von Mises stress are shown in Figure 4.

Despite the fact that an essential part of the geometries shows the maximum von Mises stress less than 11.6 MPa (this value is a destruction threshold for the pericardium used in bioprostheses [25]), some models exceed this threshold. It speaks of the initially non-optimal parameters used in designing the leaflet apparatus, which will most likely result in dysfunction of the heart valve prosthesis.

**Figure 5.**
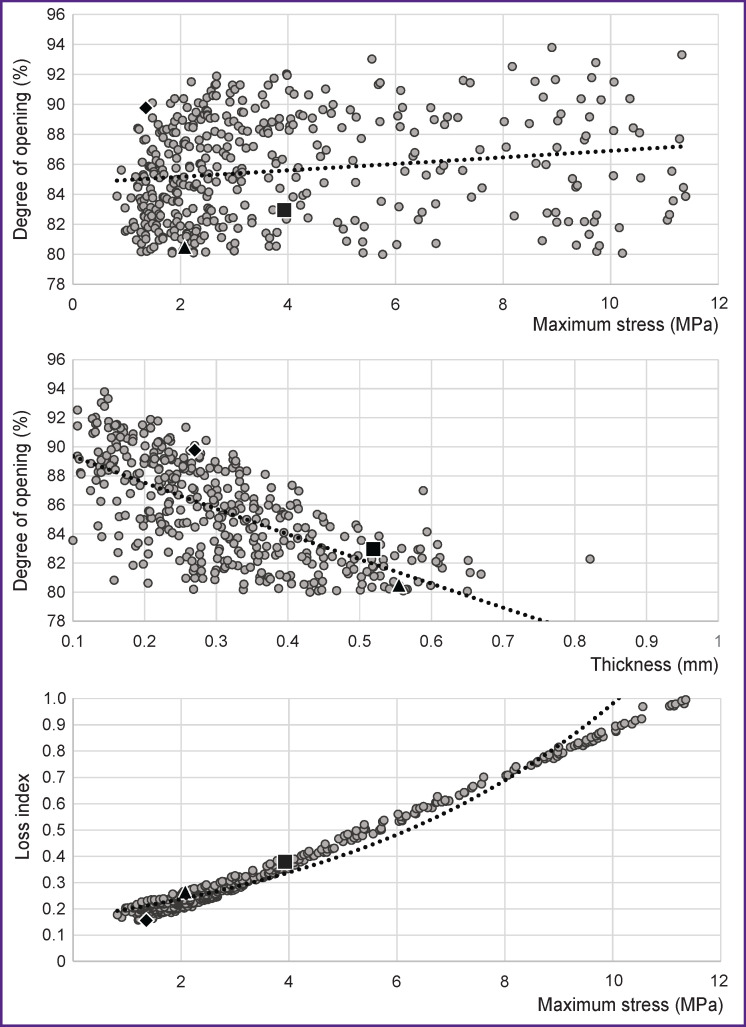
Distribution of geometries after the selection by the criteria “degree of opening” more than 80% and “maximum stress” less than 11.6 MPa Dash line — exponential trend line. Rhomb, square, and triangle mark the location of leaflet apparatus designs with 19-, 21-, and 23-mm standard sizes, respectively (see “Optimal” geometry”)

A reasonable question is: which of the obtained models gives considerable DO (more than 80% in our case) and at the same time have the peak values below the destruction threshold. To find the solution, the results obtained have been sorted using the suggested conditions. As a result, 403 models were derived with the mean DO values (85.63±3.43%) and maximum stresses (3.81±2.72 MPa). The graphs of dependences of maximum stress or DO on geometric parameters (apart from a leaflet thickness, Figure 5) do not show any evident regularity and the trend line represents a horizontal line, that is why they are not shown in Figure 5. The leaflet thickness has influenced the DO considerably. This effect may be associated with physical and mechanical properties of the pericardium used for the material models.

It should be noted that 69% of all 403 selected leaflet apparatus models have peak values below 4 MPa, the average LI=0.25 (minimal — 0.16, maximal — 0.36), which gives space for the selection of “optimal” geometries. The data presented in Figure 5 allow us to suppose that:

1. the higher the stress, the greater the degree of opening;
2. the thicker the leaflet, the lower the degree of opening and, consequently, the smaller maximum arising stresses in the leaflet apparatus.

### “Optimal” geometry

The main applied motivation for the creation of this algorithm is an automatic selection of such geometric parameters of the leaflet apparatus which provide the greatest performance — the area of opening at minimally possible stress amplitudes. Of the large set of the leaflet geometry designs selected in the process of investigation (see markers in Figure 5), 3 models (see the Table) for the 19-, 21-, and 23-mm prostheses are designated as conventionally “optimal”.

**Table:** Characteristics of “optimal” designs of leaflet apparatuses

Parameter	Prosthesis model 21 mm		
	19 mm	21 mm	23 mm
φ (°)	5.17	–26.16	–7.94
H1 (mm)	15.22	18.81	22.52
Rin (mm)	9.38	10.49	11.56
Sec (°)	119	119	119
Т (mm)	0.14	0.27	0.19
Degree of opening (%)	88.28	84.48	88.12
dmax (MPa)	3.62	1.21	1.87
Loss index	0.24	0.19	0.2

**Figure 6.**
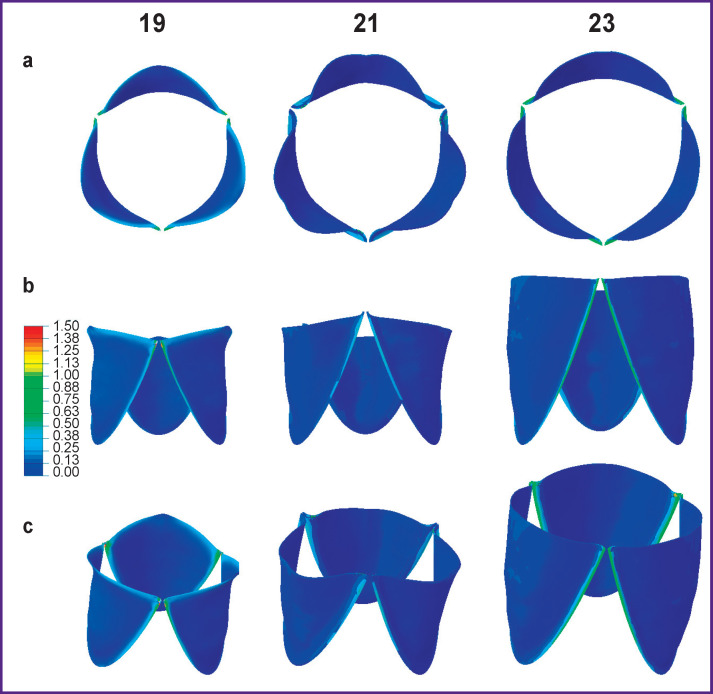
“Optimal” designs of leaflet apparatuses with 19-, 21-, and 23-mm standard sizes in the open state: (а) top view, (b) side view, (c) isometric view

Figure 6 shows “optimal” leaflet apparatuses in the closed and open state. Stresses are localized in the commissural zones, but numerical values do not exceed critical for the biomaterial model used. Qualitatively, it is seen that these models provide complete and symmetric opening with maximization of the area.

## Discussion

The algorithm of assessing the effect of geometric parameters of the heart valve prostheses on the peak stress and DO values, the most critical in selecting the optimal leaflet shapes, is presented in this work. Verification of the algorithm operation was exemplified by the opening area of the leaflet apparatus of 19-, 21-, and 23-mm Trifecta prosthesis. This step has shown the convergence of the proposed algorithm with clinical data [27-29] and manufacturer’s documentation [30].

A large number of leaflet apparatus models have been generated using various parameters. The results obtained show that in the models with greater lumen area in the open state, the arising stress increases and, in some combinations of the geometric parameters, the final design may be initially “non-viable” due to critical stresses in the valve leaflet material. However, using this algorithm at the stage of prosthesis design, it is possible to find such combinations of geometric parameters that the final item would be optimal by the two main criteria of durability in terms of biomechanics and functioning: maximal DO and minimal value of von Mises stresses. An additional benefit of this method is gaining a large bulk of information about the biomechanics of a specific design depending on the material used.

The results of this work agree with the data of other studies: a small change in one of the parameters can be critically reflected on the arising peak stresses and DO. The material of the leaflet apparatus and thickness of the leaflet itself play an important role in the quality of the prosthetic valve operation [20, 31].

### Application prospects

In the recent decade, a lot of works appeared on the investigation of polymer prosthetic heart valves [9, 32–37]. The undisputable advantage of this material is the possibility of fabricating devices of a complicated shape, which is almost impossible to achieve for their biological analogs due to cutting of the latter from the sheet biomaterial. The presented algorithm of automatic generation and investigation of the obtained leaflet apparatus models may be applied for creation of optimal designs of polymer leaflets from the standpoint of biomechanics. Since the polymer-based heart valve prostheses are not limited by cutting from the plain sheet, this approach opens new possibilities for engineers: manufacturing the products by molding, immersion (dipping) [32, 33], or 3D printing [35]. These advantages over bioprostheses make it possible to use more complex and effective designs of the leaflet apparatus, which may be selected using the presented algorithm.

### Limitations

Limitations of the described algorithm are worth mentioning. As the algorithm is a primary approximation to the complex development of the tools for engineer support in the field of designing heart valve prostheses, we used the model of one leaflet, which does not take into consideration the contacts with two adjacent leaflets in the diastolic phase. Further improvement of the algorithm will solve the task adding to the obtained results the area of cooptation and contact stress. The base of geometric and mechanical characteristics of leaflet designs may be employed as a starting point in creating the most suitable leaflet apparatuses for specific prosthetic models using a machine learning technique — the next step towards the development of the optimal geometry.

## Conclusion

The algorithm for automatic generation and evaluation of leaflet apparatuses for heart valve prostheses has been developed and verified. Its verification was performed on the basis of Trifecta bioprosthesis of three standard sizes. Of the 1517 models obtained, 278 showed the opening degree greater than 80% and maximum peak von Mises stresses below 4 MPa for the proposed model of the pericardium, which is 65% below the ultimate strength of the material. The presented designs show the capability of the algorithm to generate “optimal” leaflet apparatuses if certain known geometric parameters are set.
